# Chromoendoscopy in Combination with Random Biopsies for Patients with Pathogenic *CDH1* Mutations Undergoing Endoscopic Surveillance

**DOI:** 10.1007/s12029-022-00831-1

**Published:** 2022-05-02

**Authors:** Benjamin Ruf, Cristiana Roggia, Christopher Schroeder, Sven Mattern, Falko Fend, Thomas Klag, Martin Götz

**Affiliations:** 1 Department of Internal Medicine I (Gastroenterology, Hepatology, Infectious Diseases), University Hospital Tübingen, Tübingen, Germany; 2 Present Address: Gastrointestinal Malignancy Section, Thoracic and Gastrointestinal Oncology Branch, Center for Cancer Research, National Cancer Institute, National Institutes of Health, Bethesda, MD 20892 USA; 3 Institute of Medical Genetics and Applied Genomics, University Hospital Tübingen, Tübingen, Germany; 4 Institute of Pathology and Neuropathology, University Hospital Tübingen, Tübingen, Germany; 5 Department of Internal Medicine IV (Gastroenterology, Oncology), Klinikum Sindelfingen-Böblingen, Bunsenstrasse 120, 71032 Böblingen, Germany

**Keywords:** Hereditary diffuse gastric cancer (HDGC), *CDH1* mutation, Surveillance, Endoscopy, Chromoendoscopy

## Abstract

**Objectives:**

Germline mutations in the *CDH1*-gene are identified as the cause of 30–40% of cases of hereditary diffuse gastric cancer, an autosomal-dominant inherited cancer predisposition syndrome. Given this high risk of developing diffuse gastric cancer, carriers of a pathogenic *CDH1* germline mutation are advised to undergo prophylactic gastrectomy. For patients preferring conservative management, endoscopic surveillance is recommended. The detection of diffuse gastric cancer using white light endoscopy, however, remains challenging.

**Methods:**

Patients with pathogenic *CDH1* mutation underwent (chromo)endoscopic surveillance or endoscopy prior to surgery. Biopsies were taken at suspicious sites identified by chromoendoscopy. In addition, endoscopically normal areas were assessed with mapping biopsies. Detection rates from endoscopic biopsies (mapping vs. targeted) and gastrectomy specimen were then compared.

**Result:**

Between 11/2015 and 12/2020, ten patients from four families with a known *CDH1* germline mutation had a total of *n* = 24 endoscopies with *n* = 518 total biopsies being examined. Three patients were diagnosed with GC during the study period. These patients all had suspicious chromoendoscopic lesions (= detection rate 100%). In two of three patients who had suspicious chromoendoscopic lesions, signet cell carcinoma was also detected in mapping biopsies and multiple additional cancer *foci* were identified in the gastrectomy specimen.

**Conclusion:**

Chromoendoscopy facilitated detection of gastric carcinoma *foci* in *CDH1* mutation carriers. Chromoendoscopy identified all patients with gastric cancer, but not all cancer *foci* present in these patients. We conclude that for patients opting against prophylactic total gastrectomy, the addition of chromoendoscopy to white light could be used to enhance diagnostic reliability of endoscopic surveillance.

## Introduction

Gastric cancer (GC) is the third most common cause of cancer mortality worldwide with a total of almost 800,000 estimated deaths in 2018 [1]. Most cases of gastric cancer occur sporadically but up to 3% of patients carry familial cancer syndromes [2]. Mutations in the *CDH1* gene (OMIM:192,090) [3], encoding for the cell-to-cell adhesion molecule E-Cadherin have been identified as the most common genetic mutation associated with hereditary diffuse gastric cancer (HDGC) [4]. Approximately 40% of patients with HDGC carry heterozygous germline mutations in the *CDH1* gene [5]. Patients with a pathogenic *CDH1* germline mutation have been reported to have a cumulative life time risk of 56% (women) to 70% (men) of developing diffuse GC before the age of 80 [5]. Patients with *CDH1*-mutant gastric cancers generally carry a particularly poor prognosis [6]. Most recently, however, the risk for patients with these pathogenic *CDH1* mutations whose families do not necessarily meet strict clinical criteria for HDGC has been reported to be lower than previously assumed [7]. In addition to the high risk of gastric cancer, women who carry this mutation have a lifetime risk developing lobular breast cancer that ranges from 42 to 55% [5, 7].

Since invasive diffuse type GC is associated with a high mortality, the International Gastric Cancer Linkage Consortium (IGCLC) guidelines recommend prophylactic total gastrectomy (PTG) for carriers of pathogenic *CDH1* germline mutations. It is advised that patients typically undergo this surgery between the ages of 20 and 30 years [8]. The authors of these guidelines also recommend baseline white-light endoscopy (WLE) prior to surgery. Annual surveillance upper-GI endoscopies should be offered to patients opting against prophylactic gastrectomy or for patients who are not eligible for surgery (e.g. due to comorbidities). Multiple mapping biopsies in combination with targeted biopsies following the Cambridge protocol are recommended for proper examination [9].

During the natural course of HDGC, characteristic multifocal early lesions composed of tiny nests of signet ring carcinoma cells spread below the intact mucosa. The submucosal spread of these early cancer cells accounts for the considerable challenge of detecting tumor *foci* by traditional white-light endoscopy in early stage, asymptomatic patients. This obstacle has prompted a need to further improve detection rates of these gastric cancer *foci* [7, 10–13]. In order to enhance diagnostic performance, the implementation of chromoendoscopy, endoscopic ultrasound (EUS), auto fluorescent imaging (AFI), and narrow band imaging (NBI) has been tested [12, 14–16].

Chromoendoscopy uses the local application of biocompatible dyes to enhance contrast for the detection of mucosal abnormalities. Previous studies have yielded inconsistent results as to the diagnostic value of chromoendoscopy for *CDH1*-patients [14, 15]. These combined findings and the fact that there have been concerns about toxicities especially for Congo red dye are reflected in current guidelines that do not recommend routine use of chromoendoscopy in germline *CDH1* mutation carriers [8].

In summary, available data on the implementation of chromoendoscopy for the care of *CDH1* mutation carriers is scarce. Further studies are therefore needed to provide compelling evidence.

## Objective

To evaluate the use of chromoendoscopy in the surveillance of *CDH1* mutation carriers, we here present the data of our single-center, retrospective cohort analysis.

## Methods

### Patients, Data Collection, and Analysis

All patients with a confirmed pathogenic *CDH1* germline mutation and available data who were managed in the cancer outpatient clinic of the comprehensive cancer center (CCC) of the University Hospital Tuebingen were included. Between November 2015 and December 2020, ten patients (seven females and three males) from four families (see Table 1) with a known pathogenic *CDH1* germline mutation were assessed. All patients had previously described pathogenic *CDH1* mutations (based on the Leiden Open Variation Database http://www.lovd.nl/). All but one patient had a positive family history for gastric cancer. This patient had multigene panel testing in the setting of a multicentric lobular breast cancer diagnosis. All other mutations were identified by routine molecular testing for familial clustering of gastric cancer. Clinical HDGC criteria for genetic germline *CDH1* testing [8] were fulfilled for all of them. Mean age at data analysis was 45.4 years (range 23–69). Prophylactic total gastrectomy (PTG) was recommended for pathogen mutation carriers in accordance with the International Gastric Cancer Linkage Consortium (IGCLC) guidelines [8]. Following mandatory counselling, a baseline endoscopy was conducted prior to surgery. Alternatively, yearly endoscopic surveillance of the upper GI-tract was offered to the patients opting not to have a prophylactic total gastrectomy.

**Table 1:** Patient characteristics

**Patient**	**Age**	**Sex**	***CDH1*** **germline mutation**	**Gastric cancer**	**Histopathology**	**TNM**	**Family history**	**Number of endoscopies**	**Number of biopsies**
A	49	f	c.583C > T, p.Gln195* in exon 5	No			Negative	6	136
B	57	m	c.1792c > T, p.Arg598* in exon 12	**Yes**	Signet ring cell carcinoma, G3	ypT1b, ypN0 (0/29), L0, V0, Pn0, R0	Positive	2	33
C	33	m		No			Positive	1	15
D	69	m	c.1565 + 1G > A; p.? in intron 10	No			Positive	5	101
E	29	f	c.1137G > A, p.? in exon 8	No			Positive	3	61
F	33	f		No			Positive	2	33
G	23	f		No			Positive	1	16
H	56	f		**Yes**	Signet ring cell carcinoma, diffuse type G3	pT1a (m1), pN0 (0/39), L0, V0, Pn0, R0	positive	1	30
I	42	f	c.1565 + 1G > A, p.? in intron 10	**Yes**	poorly differentiated signet ring cell adenocarcinoma, diffuse type, G3	pT1a (m1), pN0 (0/32), L0, V0, P0, R0	Positive	2	36
J	63	f		No			Positive	1	57

**Fig. 1:**
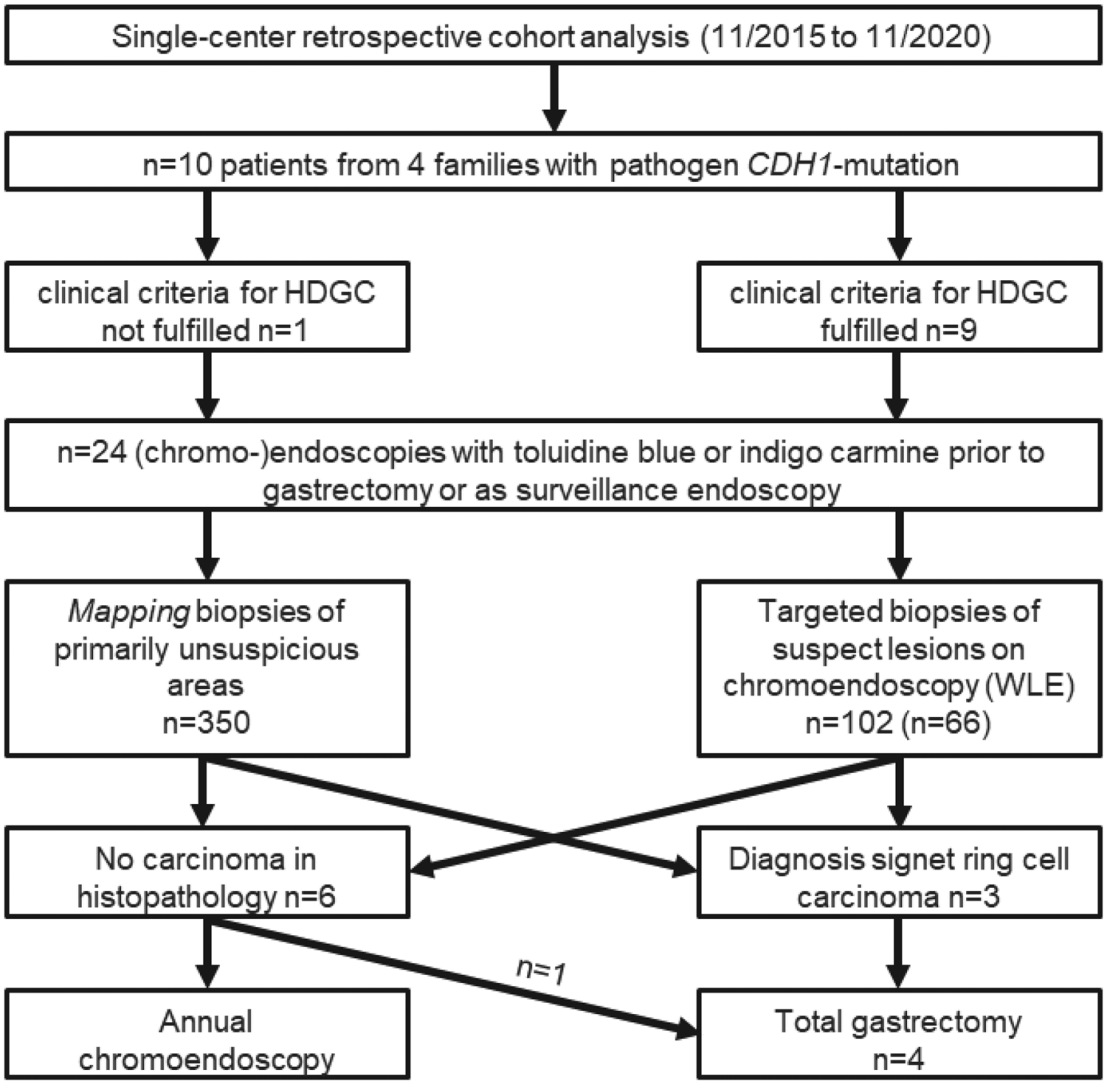
Study design

Outpatient clinic letters, family history, genomic sequencing results, endoscopy protocols, surgery protocols, and pathology reports were recorded and analyzed retrospectively. The study design is summarized in Fig. 1.

This cohort analysis was approved by the ethics committee of the medical faculty of the University of Tuebingen (project number: 216/2019BO2).

### Chromoendoscopy

Patients’ informed consent was checked prior to endoscopy and fasting was recommended for the day of the procedure. Baseline white-light endoscopy (WLE) was performed following a standardized protocol by a team of very experienced endoscopists (> 10 years of experience) under conscious sedation with propofol. High-resolution endoscopes (i-scan series, Pentax, Tokyo, Japan) were used for the examination. Chromoendoscopy of the stomach was achieved by applying indigo carmine (AminoAG, Gebensdorf, Switzerland; final concentration 0.8 mg/ml) or toluidine blue (Dr. Franz Köhler Chemie, Bensheim, Germany; final concentration 30 mg/ml) for better contrast and to unmask subtle lesions via a spraying catheter (Medwork, Höchstadt, Germany). If no obvious mucosal lesions were detectable, random mapping biopsies were obtained from the entire stomach (fundus, cardia, antrum, body). Water jet irrigation was routinely used during endoscopy.

### Pathology

Biopsies for each site were submitted in separate containers to histopathology. The specimens were fixed in formalin, paraffin-embedded and worked up according to standard diagnostic procedures. Serial sections were stained with hematoxylin and eosin, PAS, and Giemsa stain. Immunohistochemistry for pan-cytokeratin was performed, if necessary. All mapping biopsies and targeted biopsies, as well as gastrectomy specimens, were reviewed by experienced histopathologists not blinded to the gastroscopy findings. Gastrectomy specimens and detailed pathological mapping studies were correlated with the biopsies taken during endoscopy. Number of tumor *foci*, location and number of biopsies per position were noted and compared to endoscopic findings. The number of lesions, location of gastric cancer *foci*, and comparison between endoscopic specimens and gastrectomy specimens was assessed retrospectively by reviewing surgery and endoscopy reports and all pathology specimens.

## Results

### Endoscopic Findings

**Fig. 2:**
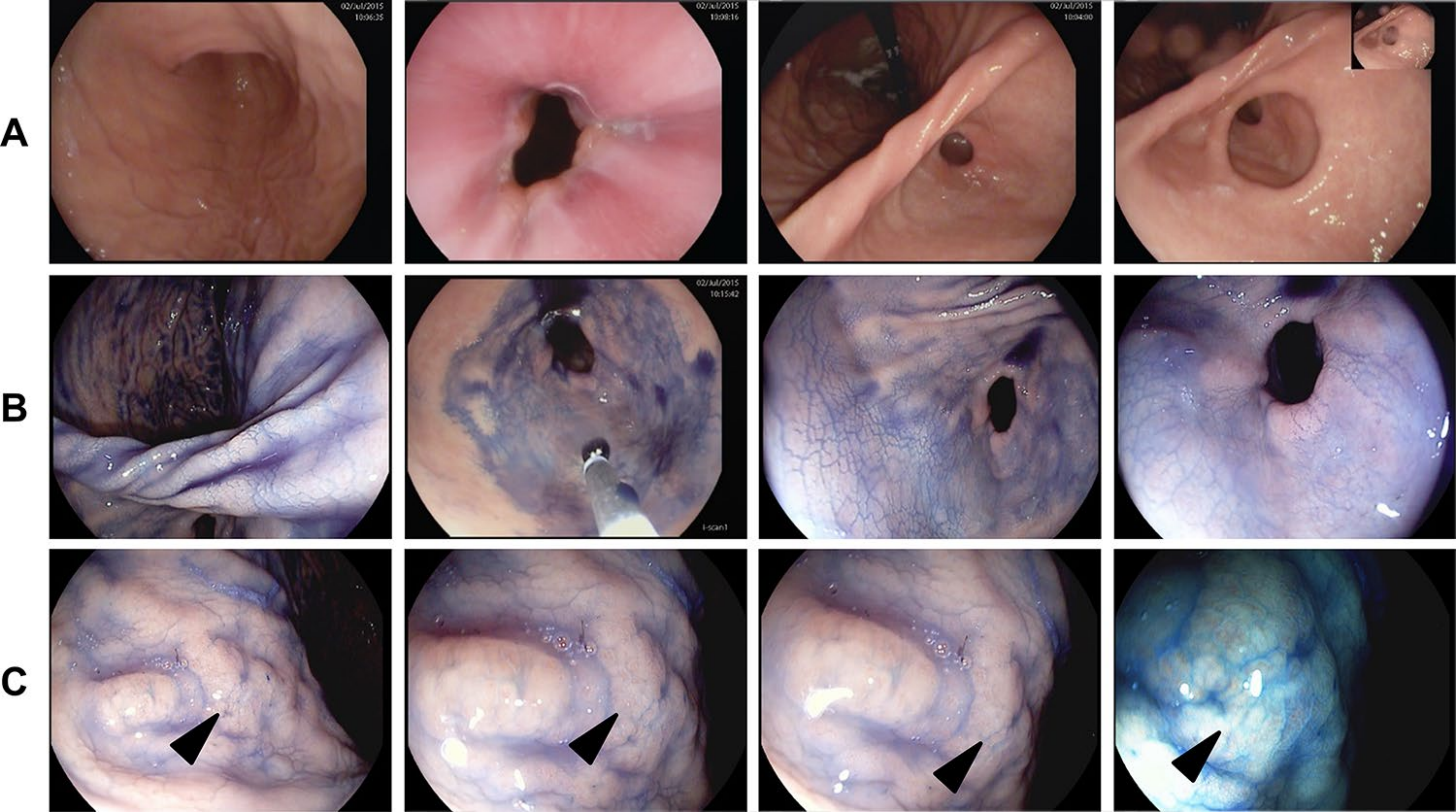
Detection of diffuse infiltrating signet cell carcinoma in a patient with a pathogen CDH1 germline mutation using chromoendoscopy. **A** Conventional white light endoscopy showed no suspicious mucosal lesions. **B** Situs after the application of indigocarmine. **C** Located at the lesser curvature, unmasking of a suspicious mucosal area the size of approximately 1 × 1 cm after the application of indigocarmine could be detected. Histopathology showed foci of a signet ring cell carcinoma

**Fig. 3:**
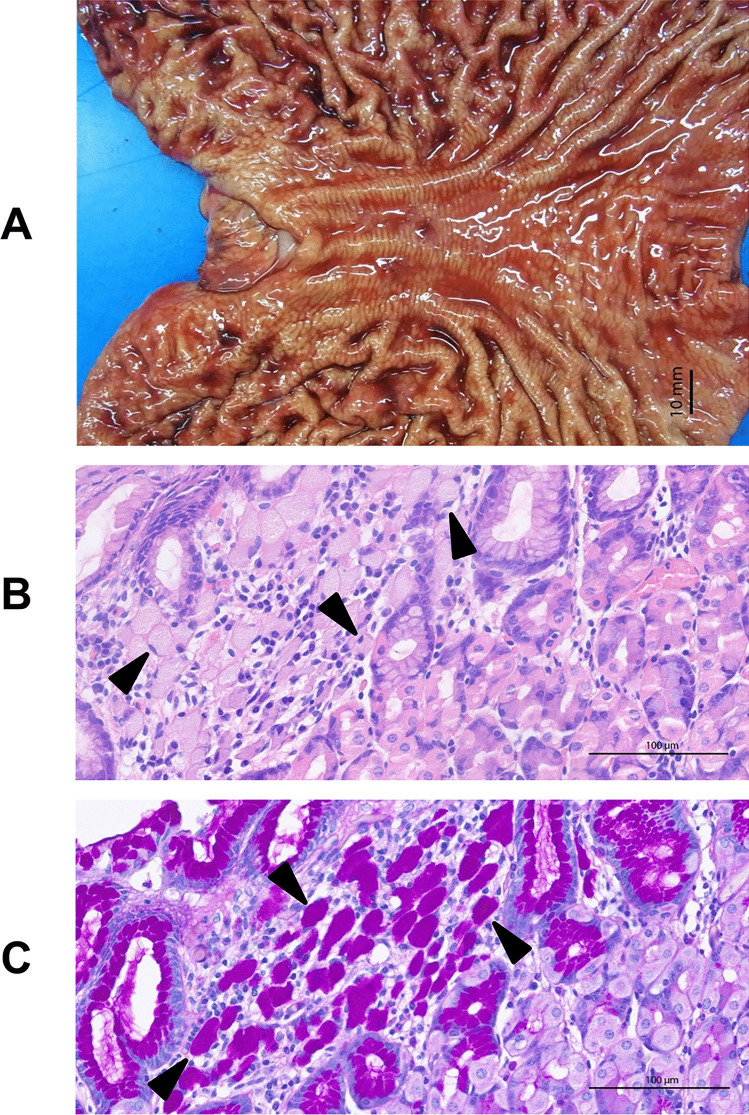
Detection of multiple foci of diffuse infiltrating signet cell carcinoma in a gastrectomy specimen (same patient as in Fig. 2). **A** Shiny, macroscopically intact mucosa in the gastrectomy specimen. **B** H&E stain reveals signet ring cell carcinoma cells below an intact mucosal layer. **C** PAS staining detects the presence of mucin in the cytoplasm of signet ring cells. Black arrows point to exemplary signet-cell carcinoma cells-

The ten patients of our cohort underwent a total of *n* = 24 upper-GI chromo-endoscopies during the study period. No adverse events occurred during the procedures. Histopathologists examined a total of *n* = 518 biopsy samples, of which *n* = 66 targeted biopsies were obtained from *n* = 36 suspect lesions detected by WLE. Another *n* = 102 were targeted biopsies from *n* = 43 lesions that were only detected after application of indigo carmine or toluidine blue. Therefore, addition of chromoendoscopy helped to unmask some additional mucosal alterations that were initially not detected in conventional white-light endoscopy. Three patients (from three different families) were endoscopically diagnosed with DGC (age at diagnosis: 18, 51, & 54 years) and were referred for subsequent total gastrectomy. For two patients, diagnosis was established at baseline (chromo)endoscopy. For the third patient, his second endoscopy 10 years after an initial white light gastroscopy (which was carried out prior to knowledge of mutational status) revealed malignant disease. A fourth patient who underwent PTG after baseline endoscopy had no pathological findings in either endoscopy or gastrectomy specimen. Interestingly, all three cancer patients had suspicious mucosal lesions detected by chromoendoscopy (= detection rate 100%). Two patients had positive biopsies in lesions only detectable after application of toluidine blue/indigo carmine stain (Fig. 2). In the third patient, a malignant lesion was detected by both WLE and chromoendoscopy. Importantly, mapping biopsies failed to detect signet ring cell carcinoma lesions seen on chromoendoscopy in one of three patients. These findings were confirmed with multiple cancer *foci* that were identified on examination of their gastrectomy specimens (Fig. 3). The third patient had one signet cell carcinoma nest that was detected only by chromoendoscopy and no additional signet ring carcinoma cell *foci* were found after TG. This patient also had neoadjuvant chemotherapy due to a suspicious enlarged lymph node that showed up on the preoperative staging CT scan. Nevertheless, there were no signs of lymph node metastasis in the gastrectomy specimen.

All patients diagnosed with gastric cancer had early or very early histological stage disease on examination of the gastrectomy specimen (#1: ypT1b; #2: pT1a (m1); #3: pT1a (m1)). No patient was found to have disease positive lymph nodes or metastatic disease. To date, no signs of recurrence in the scheduled post-operative aftercare examinations have been detected (median follow-up of 55.5 months).

The remaining six patients under surveillance had a total of *n* = 20 (chromo)endoscopies including *n* = 339 biopsies taken (including targeted biopsies from a total of *n* = 36 altered mucosal areas only identified by chromoendoscopy). For the surveillance cohort, one patient opted not to have any further surveillance endoscopies due to relevant comorbidities. The five patients who are still being monitored have a median time of surveillance at our academic center of 37.0 months (range 23–79 months) since diagnosis of *CDH1* mutation. Histopathology has not uncovered any further cases of gastric cancer to date, and none of the patients have developed clinical signs of malignant disease. The oldest patient in our cohort was 69 years old at the time of data analysis. This patient had a total of five surveillance endoscopies with no signs of GC.

## Discussion

Given the lifetime risk for *CDH1* carriers especially from families fulfilling HDGC criteria, prophylactic gastrectomy represents the current standard of care. This recommendation is the result of inadequate diagnostic and screening modalities. The value of upper-GI endoscopy for detecting early stage diffuse GC has, to date, been disappointing. Whenever signet ring cells have breached the submucosal layer, patients’ prognosis is dismal. However, the drivers of transition from indolent T1a signet ring cell carcinoma to invasive disease remain unknown [17]. In addition, genotype–phenotype correlations for *CDH1* alterations remain hard to predict [18–20]. Recent updates also report a lower risk for patients with pathogenic *CDH1* mutations whose families do not necessarily meet strict clinical criteria for HDGC [7]. In one study, Roberts et al. estimate the incidence of gastric cancer for families with pathogenic variants of *CDH1* to be 42% in male and 33% in female carriers. Another Yale-based study reported a cumulative risk of developing gastric cancer of 37.2% for men and 24.4% for women in a cohort of 113 unselected *CDH1* mutation carriers (and 476 family members) who had genetic testing due to familial clustering of various cancers. Both studies indicate that penetrance of HDGC might have been overestimated in the past, yet the risk of this aggressive malignancy remains high and should not be underestimated. Nevertheless, these results support the need for prudent surveillance strategies and stratification of patients according to personal risk.

Another challenge is finding the optimal timing for prophylactic total gastrectomy in patients with pathogenic *CDH1* germline mutations. PTG is a procedure with a postoperative morbidity that nears 100%, including weight loss, adjustments of eating habits, dumping syndrome, malabsorption, and nutritional deficiencies to name only a few [21]. Unsurprisingly, patients are reluctant when faced with the decision to undergo surgery. Factors influencing this decision include confirmatory genetic testing for *CDH1* mutation, positive biopsy results, and perceived familial cancer burden. Current guidelines recommend PTG at a young age for all pathogenic variants of *CDH1*. Due to the relatively young age of our cohort (mean age 45.4), it seems likely that more patients might develop DGC over the coming years (previously described mean age at diagnosis 46.7 years [7]) and early detection will be critical for improved prognosis.

Thorough white-light endoscopy with both random and targeted biopsies was reported to identify 63.6% *CDH1* mutation carriers and 28.6% non-pathogenic mutations of signet ring cells in 29 patients fulfilling criteria for HDGC [12]. A recent study examining 20 carriers of *CDH1* germline mutations who did not fulfill family history criteria for HDGC reported that while none had suspicious diagnostic findings on conventional endoscopy, 12 showed signet ring carcinoma cells on histology of gastrectomy specimens or random biopsies [22]. In a relatively large surveillance study on 54 *CDH1*-mutated patients (and 31 *CDH1*^−^), 36 patients were diagnosed with signet ring carcinoma including 15 that had positive histological findings from targeted biopsy sampling [11]. An even more rigorous WLE sampling protocol with a systematic visualization and biopsy approach (the so-called Bethesda protocol) has recently shown to increase detection rates as compared to the Cambridge protocol (15% vs. 34%) [13]. One limitation of the current study is that the minimum number of biopsies per patient as per Cambridge protocol was not reached in our real-life setting study (average of 21.5 biopsies obtained vs. minimum of 24 biopsies per patient recommended). Also, examination time for WLE was not noted in clinical practice and could, thus, not be evaluated in the current retrospective study to see if the recommended 30 min per examination were reached.

Our findings demonstrate that chromoendoscopy could potentially further enhance detection rate of diffuse gastric carcinoma in patients with pathogenic *CDH1* mutations compared to traditional white-light endoscopy. In our cohort, all patients who developed DGC to date had suspicious findings on chromoendoscopy. Sensitivity for overall tumor detection for these patients was technically 100%. Naturally, it cannot be excluded that signet ring carcinoma cell *foci* remain undetected thus far in patients who are undergoing surveillance. These findings are consistent with earlier retrospective cohort analysis of 33 *CDH1* mutation carriers in New Zealand [14]. Shaw et al. also conducted 93 chromoendoscopies (using Congo red and methylene blue) and detected 23 signet ring cell carcinoma *foci* (in 10 patients) otherwise not detectable by white-light gastroscopy. Nevertheless, the detection rate for additional neoplastic *foci,* which were not apparent during endoscopy, was also considerably lower in this study. As in our study, these foci were only detectable in gastrectomy specimens. Importantly, detection rates for signet ring carcinoma cell *foci* < 2 mm were low, a finding shared by a prospective study of 8 German patients [15]. Hüneburg et al. reported that chromoendoscopy in addition to mapping did not reveal further cancerous lesions, although 6/8 patients had multiple *foci* of gastric carcinoma identified in the surgical specimens. All of these lesions were < 2 mm. Of note, the number of DGC *foci* per patient differed significantly between the New Zealand and German cohorts (157 vs. 4.5 *foci*/patient), partially explaining the contradictory findings. Low detection rate for total number of signet ring carcinoma *foci* found in gastrectomy specimens is an observation that also applies to our cohort analysis, where two out of three patients with GC had multiple additional signet ring carcinoma cell *foci* identified only after PTG. Random mapping biopsies following the Cambridge (or better Bethesda) protocol [9] should therefore still be additionally performed to maximize diagnostic safety for patients undergoing endoscopic surveillance.

As previously stated, the conclusions that can be drawn from this small single-center cohort study are limited. First, HDGC due to *CDH1* germline mutation is a rare disease, and therefore, this study lacks a representative control group. Second, knowledge about the natural history of HDGC in *CDH1*-mutated patients is lacking but is currently being investigated (NCT03030404). The genotype–phenotype correlation has not yet been fully established and the timeline of progression from in situ signet ring cell carcinoma to infiltrating gastric cancer remains to be established. As such, the clinical relevance of small tumor *foci*, undetectable by (chromo-)endoscopy, has not yet been fully elucidated. The recommendation of prophylactic total gastrectomy should remain the standard of care at this time, despite the dreadful prospect and high mortality that patients are faced with. Patients may be more willing undergo surgery if early signet ring carcinoma could be detected through (chromo-)endoscopically-guided biopsies. Nevertheless, the life-changing elements of a PTG and the risk of developing metastatic disease [10] must be carefully weighed on an individual basis and informed consent must be ensured.

Here, we propose the implementation of chromoendoscopy for endoscopic GC surveillance of patients with *CDH1* mutations to enhance diagnostic safety. Other technologies to enhance diagnostic sensitivity of upper-GI endoscopy for *CDH1* surveillance such as confocal endoscopic microscopy are currently being evaluated (ClinicalTrials.gov Identifier: NCT03648879) [23]. Given the predominantly submucosal growth of cancer foci in *CDH1* mutation carriers, additional endoscopic ultrasound (EUS) might offer technological advancement. Nevertheless, accuracy, sensitivity, and positive predictive value and negative predictive value of EUS in combination with fine needle aspiration (FNA) might be limited to detect microscopic, multicellular cancer cell nests. Not surprisingly, a recent study demonstrated limited utility for diagnostic purposes in CDH-1 mutation carriers [24]. Beyond technological advancement, it has recently been demonstrated that an even more rigorous, systematic sampling following the so called “Bethesda” protocol, could improve accuracy of endoscopic surveillance beyond the previous consensus following the Cambridge protocol [13].

Further clinical testing of chromoendoscopy in a larger patient cohort in a prospective manner following a standardized protocol should be conducted in order to fully elucidate the potential as well as the limitations of this approach.

## Conclusion

The use of chromoendoscopy by an experienced examiner following conventional high definition white light gastroscopy might facilitate detection of early gastric carcinoma *foci*. Thus, for patients opting to postpone or forego prophylactic total gastrectomy, additional chromoendoscopy could potentially be used to better guide optimal timing and could have the potential to enhance diagnostic efficacy for patients under endoscopic surveillance.

## Abbreviations

AFI: Auto fluorescent imaging
CCC: Comprehensive cancer center
DGC: Diffuse gastric cancer
EUS: Endoscopic ultrasound
GC: Gastric cancer
HDGC: Hereditary diffuse gastric cancer
H&E: Hematoxylin and eosin
IGCLC: International Gastric Cancer Linkage Consortium
NBI: Narrow band imaging
NCI: US National Cancer Institute
PAS: Periodic acid–Schiff
PTG: Prophylactic total gastrectomy
WLE: White-light endoscopy
